# Sufficient Standardization? Evaluating the Reliability of an Inertial Sensor (Beyond^TM^) for Ankle Dorsiflexion After a Brief Familiarization Period

**DOI:** 10.3390/sports13120447

**Published:** 2025-12-11

**Authors:** Giacomo Belmonte, Alberto Canzone, Marco Gervasi, Eneko Fernández-Peña, Angelo Iovane, Antonino Bianco, Antonino Patti

**Affiliations:** 1 Sport and Exercise Sciences Research Unit, Department of Psychology, Educational Science and Human Movement, University of Palermo, 90144 Palermo, Italy; giacomo.belmonte@unipa.it (G.B.); alberto.canzone98@gmail.com (A.C.); angelo.iovane@unipa.it (A.I.); antonino.bianco@unipa.it (A.B.); 2 Department of Neuroscience, Biomedicine and Movement, University of Verona, Via Felice Casorati 43, 37131 Verona, Italy; 3 Department of Biomolecular Sciences, Division of Exercise and Health Sciences, University of Urbino Carlo Bo, 61029 Urbino, Italy; marco.gervasi@uniurb.it; 4 Department of Physical Education and Sport, University of the Basque Country UPV/EHU, 01007 Vitoria-Gasteiz, Spain; eneko.fernandezp@ehu.eus

**Keywords:** CAI, ankle sprain, IMU, biomechanics, physical activity, sport performance

## Abstract

(1) Background: Ankle joint range of motion is recognized as abnormal in individuals with ankle sprains and Chronic ankle instability (CAI), especially in the dorsiflexion movement. This research investigated the test–retest and inter-rater reliability of the Motustech Beyond IMU for dorsiflexion movement following only one hour of rater training and familiarization. (2) Methods: In total, 62 subjects were evaluated for the inter-rater reliability and test–retest with a one-week interval. The intraclass correlation coefficient (ICC), along with the Concordance Correlation Coefficient (CCC), was determined for each test of reliability. Standard error of measurement, coefficients of variation, limits of agreement (LoA) and minimal detectable change (MDC) were used for the measurement error analysis. (3) Results: Test–retest reliability was ranked excellent (ICC = 0.949) and very high (CCC = 0.897) for both ankle dorsiflexion measurements. On the other hand, Inter-Rater reliability was evaluated as good (ICC = 0.881–0.906) and very high (CCC = 0.783–0.811). However, the measurement error analysis showed poor absolute agreement (LoA), indicating that the resulting measurement variability is considered clinically unacceptable for high-precision applications. (4) Conclusions: Beyond Inertial demonstrated excellent test–retest reliability for ankle dorsiflexion movements, although measurement error analysis showed considerable absolute error. Consequently, it may be considered a reliable tool for single-rater monitoring of ankle dorsiflexion ROM in non-clinical settings such as general physical activity and amateur sports. Future research should investigate its potential role in injury prevention contexts.

## 1. Introduction

Chronic ankle instability (CAI) and ankle sprains are among the more frequent lower extremity injuries in daily life and amateur sports activities, with a prevalence ranging from 25% to 46% [1,2]. An ankle sprain often causes impaired proprioception, instability, and difficulty walking, followed by symptoms such as weakness, pain, and decreased joint stability [3]. Repeated ankle sprains or inadequate recovery are highly associated with CAI development, showing impairments in the quality of life [4,5]. Indeed, CAI represents a clinical disorder, often followed by recurrent incidents of lateral ankle sprains (LAS) [6]. The major risk factors for ankle sprains and CAI are categorized as intrinsic factors, including body composition, age, gender, history of previous injuries, muscle strength, and postural balance, followed by extrinsic risk factors, such as participation in a specific type of sport [7]. Regarding the type of sport, in a recent study, Figlioli et al. assessed the applicability of a 9-item questionnaire established to evaluate ankle pain, ankle instability, ankle sprains, and the period of recovery after repeated ankle sprains in volleyball players [8]. CAI’s updated model has eight main components: sensory-perceptual impairment, pathomechanical impairment, personal factors, primary tissue injury, motor-behavioral impairment, interactions between components, spectrum of clinical outcomes, and environmental factors [9]. In addition, individuals with CAI often exhibit abnormalities in ankle range of motion (ROM), especially in the dorsiflexion movements [10,11]. Sufficient dorsiflexion range of motion is essential for achieving a stable position of the ankle joint, and a reduction in this range compromises stability during functional movements [12]. The relevance of ankle dorsiflexion movement has been widely recognized in the literature, especially in subjects with CAI and recurrent ankle sprains [13,14]. Moreover, scientific literature provides evidence of exercise interventions aimed at increasing ankle joint ROM [15,16]. Stretching is known as an effective method to enhance ankle joint ROM [17,18]. Specifically, static stretching protocols in the calf area with a total duration of between 15 and 30 min have shown significant long-term effects on ankle dorsiflexion ROM [19]. In addition to stretching, several recent studies have examined the effects of foam rolling (FR) on joint mobility [20,21]. However, as reported by Kelly and Beardsley, the joint ROM of the ankle in acute cases appears to increase significantly for a limited time of up to 20 min [22]. Nevertheless, the applicability of the FR in CAI subjects has already been studied, but its medium- to long-term effects on ankle dorsiflexion are unclear and seem to be related to the weeks of intervention [21,23]. Therefore, accurately and reliably measuring joint range of motion is essential for specialists and professionals in implementing their diagnostic and therapeutic strategies. There are several methods for assessing and measuring ankle ROM [24,25,26]. The goniometer represents a simple and inexpensive objective tool, but instruments such as cameras and wearable devices have been developed recently [27,28,29,30]. Furthermore, this type of instrument requires considerable experience from raters to ensure measurement reproducibility [24,25,26]. Inertial measurement units (IMUs) represent a combination of three sensors: an accelerometer, magnetometer, and gyroscope, able to measure acceleration, angular velocity, and the magnetic field vector with direction [31,32]. These instruments can be used to evaluate several joints, including the elbow, spine, knee, and ankle [33,34]. They facilitate the easy and reproducible measurement of body segment orientation by analyzing data related to acceleration and angular velocity [35]. The use of these IMUs is not error-free during data acquisition. The main problem with inertial sensors is related to drift and distortion errors [35,36]. However, these limitations can be mitigated by the IMU system, which, by combining its three sensors, can deliver precise data [37]. Recently, the scientific literature has recognized the reliability and validity of several IMUs in different joints for the movements they perform in people with and without injuries [38,39]. The BEYOND Inertial, Motustech Sport & Health Technology, is a wearable IMU sensor that can analyze joint mobility and thus ROM quickly, easily, and accessibly in most environments, in a non-invasive manner. However, the reliability of this specific device has not been previously established in the scientific literature. Furthermore, few studies assess ankle function using an inertial sensor exclusively for ankle dorsiflexion motion. Therefore, the current study aimed to assess the test–retest and inter-rater reliability of the BEYOND inertial system after only one hour of familiarization by the raters for ankle ROM joint dorsiflexion movements.

## 2. Materials and Methods

### 2.1. Study Design and Procedure

The study design, sample selection, and statistical analysis were conducted in accordance with the GRRAS guidelines [40], which recommends transparent reporting of reliability studies. Moreover, for the sample recruitment number, the methodological quality of systematic reviews of studies on measurement properties was considered [41]. Data were collected by two different raters. Before the official measurements, participants received thorough instructions on the protocol to ensure movements were performed as intended. Reliability measurements focused on dorsiflexion movement of both the right and left ankle joints within the sagittal plane. Participants were seated on a massage table with the popliteal fossa in full contact with the lateral side. They were instructed to perform the maximal ankle dorsiflexion movement starting from a relaxed foot position, taking care not to extend the knees or raise the heels during the execution. The starting position was the same for all participants. The raters observed the movement execution and immediately excluded any incorrectly performed trials. Each participant performed three repetitions per ankle. Before each measurement, the sensor was attached by the rater, who identified the midpoint of the foot and secured the Beyond inertial sensor using a tear band. The first measurement was performed by Rater 1 for each participant. After this examination, the Beyond was removed and repositioned by Rater 2 for the inter-rater reliability. To verify test–retest reliability, participants were assessed twice, one week apart, by the same rater at the same time of day. Participants were asked to maintain the same routines the day before the assessment. On the first day, the inter-rater reliability was administered by both raters (see Table 1).

### 2.2. Raters and Familiarization Protocol

The measurements were carried out by two raters, a Ph.D. student and a Research Fellow, after training and standardization of the Beyond inertial sensor on the ankle joint for about one hour. During the training and familiarization session, the raters initially explored the specific inertial sensor software on a personal computer. Once they had identified the correct location of the dashboard for joint ROM assessment, they focused on standardizing the inertial sensor on the ankle joint. The different tear strips provided with the sensor were tested to find the most suitable for assessing ankle movement. Subsequently, once identified, it was positioned in the center of the foot to exclude toe movement during measurements. To ensure the full range of ankle dorsiflexion could be evaluated, raters standardized the starting position for all participants, beginning from a relaxed foot position.

### 2.3. Participants

The Participants of the study were exclusively healthy university students, voluntarily recruited by the Center for Research in Sport and Exercise Science at the University of Palermo. The measurements were conducted between 10:00 and 12:30 a.m. in the “Laboratory of Posture and Biomechanics”, a research center with a regulated temperature (23°). This study was part of regular training at the educational pole of the University of Palermo. A Ph.D. student interviewed participants to collect general information and apply the study’s inclusion and exclusion criteria. Inclusion criteria were participants between the ages of 18 and 30 years with signed informed consent, approved by the Bioethics Committee of the University of Palermo (n. 94/2022-Prot. 70310; 4 July 2022) according to the principles of the Declaration of Helsinki. Exclusion criteria included participants with CAI, an ankle sprain within the past six months, or those who had undergone ankle or knee joint surgery.

### 2.4. Instrument

**Figure 1:**
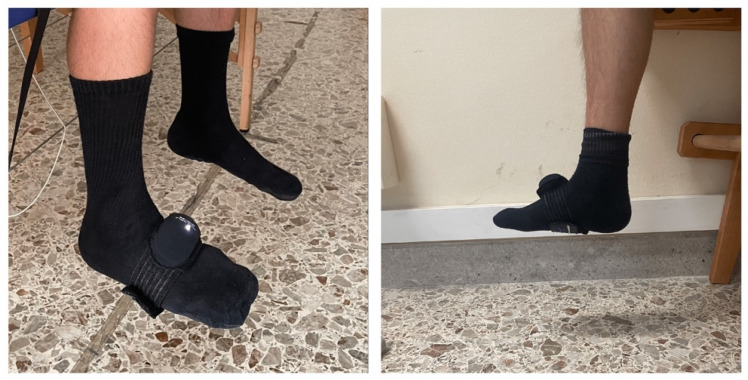
Instrument setting of Beyond Inertial on the ankle joint.

The BEYOND Inertial (Motustech—Sport & Health Technology, Roma, Italy) is an inertial sensor able to assess joint mobility (ROM), jump, balance (using standardized tests in different situations and on different support surfaces), and power analysis (analyzing the force, speed, power, and smoothness of the movement of a load). It allows internal imaging sampling up to 1000 Hz and connects to software via Bluetooth. It has the function of a tri-axial gyroscope with programmable FRS of ±250 dps, ±500 dps, ±1000 dps, and ±2000 dps; a tri-axial accelerometer with programmable FRS of ±2 g, ±4 g, ±8 g, and ±16 g; and a tri-axial compass with a range of ±4900 μT. Figure 1 shows the Beyond Inertial and measurement setting.

### 2.5. Statistical Analysis

Descriptive statistics were recorded in Excel (version 16.32) for each participant before analysis. Then, the data were uploaded to Jamovi software (version 2.3.28) and GraphPad (version 8.02) to perform statistical analysis. The maximal ROM value was used for both ankle dorsiflexion movements. The results were reported as means and standard deviations. Inter-rater and test–retest analyses were performed to evaluate the reliability of the Beyond inertial sensor. Firstly, A within-subjects repeated measures ANOVA was performed to examine the test–retest reliability of rater 1. The analysis evaluated the evolution over time, with gender as a between-subject factor, to assess statistical differences between the measurements on Day 1 and Day 2 with one week apart. Secondly, an average intraclass correlation coefficient (ICC) along with a Concordance Correlation Coefficient (CCC), including 95% confidence intervals, was calculated for test–retest reliability and inter-rater reliability. Specifically, two-way fixed effects with average consistency Intraclass Correlation Coefficients (ICC 3,k) were calculated to determine the reliability of each test [40,42]. This ICC model was selected because the study used a specific, fixed group of evaluators (for inter-rater reliability) and a fixed number of tests repeated at different times (for test–retest reliability) [40,42]. ICCs were interpreted as follows: <0.50 = poor; between 0.50 and 0.75 = moderate; 0.75–0.90 = good; and >0.90 = excellent [42]. Instead, CCC values were interpreted as: <0.1 = trivial; between 0.1 and 0.29 = small, 0.3–0.49 = moderate, 0.5–0.69 = high, 0.7–0.89 = very high, and >0.9 = practically perfect [43]. Thirdly, the standard error of measurement (SEM) was calculated to assess the error component of measurements, along with the coefficients of variation (CV), the limits of agreement (LoA) and the minimal detectable change (MDC). The MDC was calculated using the following formula:

MDC = 1.96 × SEM × √2

Bland–Altman plots of each reliability test were presented to examine the limits of agreement [44]. The effect size (η^2^_p_) was interpreted as follows: less than 0.01 as a small effect; between 0.02 and 0.1 as a moderate effect; and greater than 0.1 as a large effect [45]. The significance level was set at *p*  ≤  0.05 for all analyses.

## 3. Results

Sixty-two healthy participants joined the study. Specifically, forty-five males and seventeen females were analyzed. The group reported a mean age of 21.4 (±2.88), a mean weight of 68.6 (±13.82), and a mean height of 172.3 (±10.81).

### 3.1. Inter-Rater Reliability

**Figure 2:**
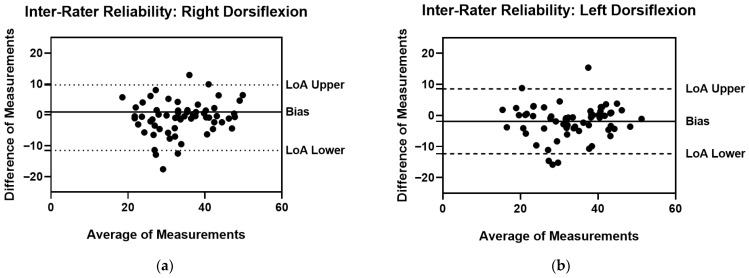
Inter-Rater Reliability Bland–Altman plots with Bias, LoA Lower and LoA Upper (**a**) Right Dorsiflexion ROM Measurements; (**b**) Left Dorsiflexion ROM Measurements.

Table 2 presents the inter-rater reliability for the right and left ankle dorsiflexion ROM. The Inter-Rater reliability of Right Dorsiflexion ROM showed a very high CCC (0.783) along with a good ICC (0.881). Instead, the Inter-Rater reliability of Left Dorsiflexion ROM recorded a very high CCC (0.811) and an excellent ICC (0.906). However, the measurement error analysis revealed elevated Limits of Agreement (LoA) and Minimal Detectable Change (MDC) for both measurements, despite good ICC and optimal CCC values. This high difference compromises the overall inter-rater reliability, particularly in a clinical setting. In this regard, the limits of the agreement are also presented through the Bland–Altman plots in Figure 2.

### 3.2. Test–Retest Reliability

**Figure 3:**
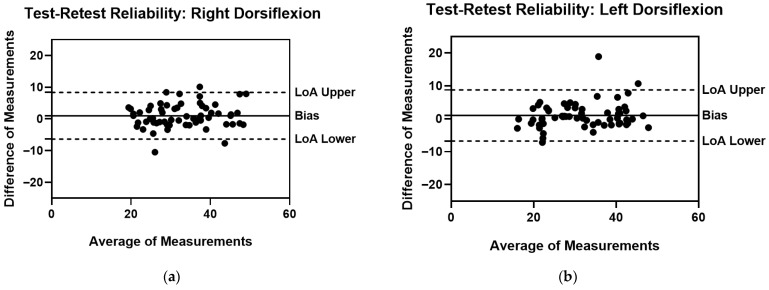
Test–retest Reliability Bland–Altman plots with Bias, LoA Lower and LoA Upper (**a**) Right Dorsiflexion ROM Measurements; (**b**) Left Dorsiflexion ROM Measurements.

Repeated measures ANOVA did not reveal statistically significant differences between test and retest measurements (*p* > 0.05). Specifically, the analysis showed no interaction determined by time on rater’s measurements for right dorsiflexion (F = 0.603; *p* = 0.440; η^2^_p_ = 0.010) and left dorsiflexion (F = 3.80; *p* = 0.056; η^2^_p_ = 0.060). Moreover, the test–retest reliability for the right and left ankle movements is presented in Table 3. Similarly, the test–retest ROM of the right dorsiflexion, together with the ROM of the left dorsiflexion, showed a very high CCC (0.897) and an excellent ICC (0.949). Nevertheless, the test–retest analysis also revealed high LoA and MDC values, although the magnitude was lower than that observed in the inter-rater reliability. Even for test–retest reliability, Bland–Altman plots are used to illustrate the statistical measurement error analysis, as shown in Figure 3.

## 4. Discussion

This study was designed to explore the reliability of a specific IMU on ankle dorsiflexion measurements in the sagittal plane through only one hour of rater training. The results demonstrated that Beyond Inertial constitutes a valid and reproducible tool for a single rater to measure ankle dorsiflexion ROM, even with a limited training and familiarization period. To confirm this, test–retest reliability analysis was conducted to investigate the influence of time on the measurements, thereby ensuring that observed variation was not attributable to measurement or random errors stemming from mechanical inconsistencies [46]. The repeated measures ANOVA did not reveal any statistically significant differences between the two measurements conducted by the same rater one week apart (*p* > 0.05). On the contrary, ICCs showed excellent values for both ankle dorsiflexion measurements (0.949), along with very high CCC values (0.897). Measurement error analysis showed acceptable CV parameters (<15%), as shown also in Bland–Altman plots [47]. These results indicate that the Beyond Inertial system could be a valuable and easy-to-use tool for a single operator to record ankle ROM. Its reliability, achieved with only a minimal familiarization period, supports its integration alongside other measurement tools currently available in the literature [48,49,50]. However, an analysis of the absolute measurement error is necessary for clinical interpretation. The LoA for test–retest was wide, ranging approximately from −6.35° to 8.34° for the right ankle and from −6.75° to 8.76° for the left ankle. Furthermore, the calculated MDC is the minimum amount of change required to exceed this measurement error [42]. This relatively large MDC means that small, but potentially clinically relevant, changes in ankle dorsiflexion cannot be reliably detected by a single rater over time using this device and protocol. So, while the relative reliability (ICC and CCC) is excellent, the error (LoA and MDC) may still limit the utility of the Beyond Inertial for detecting subtle changes in a clinical setting. Regarding inter-rater reliability, the analysis assessed the reproducibility of ankle dorsiflexion measurements between two independent raters. The ICC values demonstrated measures ranging from good to excellent for each dorsiflexion movement (0.881–0.906) [42]. Concurrently, the Concordance Correlation Coefficient (CCC) values also indicated very high reliability, ranging from 0.783 to 0.811. However, the lowest observed CCC value (0.783), specifically for the right dorsiflexion measurement, should be noted, as it approaches the accepted lower threshold of 0.69 [43]. Crucially, the measurement error analysis for inter-rater reliability yielded results that contradict the favorable ICC and CCC findings. Despite the good reliability indicated by these coefficients, the Limits of Agreement (LoA) were poor, showing a wide and clinically significant range of error: from −8.56° to 12.32° for left ankle dorsiflexion and from −11.51° to 9.67° for right dorsiflexion. This level of error is clinically intolerable, suggesting that the one-hour training protocol was likely insufficient to establish adequate inter-rater reliability for clinical environments. To our knowledge, few studies have specifically examined the reliability of an IMU for ankle joint measurements. For example, a recent study by Bauer et al. evaluated the reliability using a two-segment IMU-based foot model for gait analysis [51]. The authors demonstrated the efficacy of a system involving multiple IMUs on various joints to evaluate the ROM of kinematics in gait analysis, including on the ankle [51]. However, no reliability related to individual joints is reported in the study [51]. On the other hand, other reliability studies were conducted on other joints with similar IMU systems to investigate the reproducibility of joint ROM measurements during specific movements. In the study conducted by Santospagnuolo et al., the GYKO inertial sensor was used to analyze elbow flexion-extension movement in the sagittal plane through intra-rater reliability and inter-rater reliability [52]. Specifically, the ICC was calculated, demonstrating good to high inter- and intra-rater reliability parameters (0.859–0.942) [52]. Similarly, Hamersma et al. evaluated the same IMU for flexion, extension, and left and right lateral flexion movements of the lumbar back ROM [53]. The authors demonstrated good to excellent inter-rater reliability through ICC values ranging from 0.82 to 0.94. In addition, measurement error analysis was evaluated using SEM and LoA. In line with the results of our study, LoA was found to be higher in inter-rater reliability than the other statistical tests of IMU reliability [53].

### Limitations

Despite the great practical implications, this study has several limitations. Initially, a randomized trial was not conducted for participants or raters. On the other hand, the measurements were limited to ankle dorsiflexion without considering the other ankle movements, such as plantar flexion, inversion, or eversion. Furthermore, the reliability of the inertial sensor was not evaluated during complex ankle movements such as jumping, walking, running, and squatting. Moreover, the study was conducted in a controlled laboratory with healthy university participants rather than in a clinical setting with ankle sprain or CAI participants. So, the reliability of Beyond should be investigated further in different environments and with different participants to explore its exact prevention potential.

## 5. Conclusions

In conclusion, the relative reliability (ICC/CCC) of the Beyond Inertial system for measuring ankle dorsiflexion in the sagittal plane was excellent for a single rater, achieved after only a single hour of training. This finding supports the system’s use as a reproducible tool for monitoring ankle ROM by one operator in contexts like general physical activity and amateur sports. However, a comprehensive analysis of the absolute measurement error reveals significant limitations for high-precision clinical applications, where a few degrees of movement can be crucial. In addition, the analysis of inter-rater reliability indicated that a one-hour training protocol was insufficient for this purpose. However, it still provided valuable insights into the sensor’s overall reliability. Therefore, despite the sensor’s internal consistency, the current protocol is unsuitable for clinical use and requires thorough and rigorous training to establish its clinical applicability.

## Figures and Tables

**Table 1:** Test protocol to measure Inter-Rater and Test–retest Reliability.

Inter-Rater Reliability	Test–Retest Reliability
Rater 1 (Day 1)	Rater 1 (Day 1)
Rater 2 (Day 1)	Rater 1 (Day 2)

**Table 2:** Inter-Rater Reliability.

Movement	Rater 1	Rater 2	Inter-Rater Reliability				Measurement Error				
	Mean ± SD	Mean ± SD	CCC	95% CI (Lower–Upper)	ICC	95% CI (Lower–Upper)	CV (%)	LoA Lower	LoA Upper	SEM	MDC
Dorsiflexion Right °	33.5 ± 8.71	34.5 ± 7.84	0.783	0.666–0.862	0.881	0.818–0.922	11.23	−11.51	9.67	3.8205	10.59
Dorsiflexion Left °	32.3 ± 9.3	34.2 ± 8.9	0.811	0.708–0.881	0.906	0.857–0.939	11.33	−8.56	12.32	3.7662	10.44

° = angles in degrees; SD = Standard Deviation; CCC = Concordance Correlation Coefficient; ICC = Interclass Correlation Coefficient; CI = Confidence Interval; CV = Coefficients of Variation; LoA = Limits of Agreement; SEM = Standard error of Measurement; MDC = Minimal Detectable Change; All ICC have values of *p* < 0.01.

**Table 3:** Test–retest Reliability.

Movement	Test	Retest	Test–Retest Reliability				Measurement Error				
	Mean ± SD	Mean ± SD	CCC	95% CI (Lower–Upper)	ICC	95% CI (Lower–Upper)	CV (%)	LoA Lower	LoA Upper	SEM	MDC
Dorsiflexion Right °	33.5 ± 8.71	32.5 ± 8.29	0.897	0.835–0.936	0.949	0.922–0.967	8.02	−6.348	8.341	2.6497	7.34
Dorsiflexion Left °	32.3 ± 9.3	31.3 ± 8.6	0.897	0.836–0.936	0.949	0.922–0.966	8.8	−6.75	8.76	2.7989	7.76

° = angles in degrees; SD = Standard Deviation; CCC = Concordance Correlation Coefficient; ICC = Interclass Correlation Coefficient; CI = Confidence Interval; CV = Coefficients of Variation; LoA = Limits of Agreement; SEM = Standard error of Measurement; MDC = Minimal Detectable Change; All ICC have values of *p* < 0.01.

## Data Availability

The data presented in this study are available on request from the corresponding author due to privacy.
